# Numerical treatment and global error estimation for thermal electro-osmosis effect on non-Newtonian nanofluid flow with time periodic variations

**DOI:** 10.1038/s41598-023-41579-3

**Published:** 2023-09-08

**Authors:** O. S. Ahmed, N. T. Eldabe, M. Y. Abou-zeid, O. H. El-kalaawy, S. M. Moawad

**Affiliations:** 1 Department of Mathematics and Computer Science, Faculty of Science, Beni-Suef University, Beni-Suef, Egypt; 2 Department of Mathematics, Faculty of Education, Ain Shams University, Heliopolis, Cairo Egypt

**Keywords:** Applied mathematics, Mechanical engineering

## Abstract

The essential purpose of this study is to discuss the impact of time-periodic variations on mixed convection heat transfer for MHD Eyring-Powell nanofluid. The fluid flows through a non-Darcy porous medium over an infinite vertical plate. The effects of viscous dissipation, Ohmic dissipation, electro-osmosis force, heat source, thermal radiation, Dufour feature, and chemical reaction are presumed. The system of partial differential equations which governs the problem is transformed into a system of non-linear algebraic equations and then an explicit finite difference approach is espoused to solve these nonlinear algebraic equations. The numerical results for the velocity, temperature, and nanoparticles concentration distributions are computed and displayed through a set of graphs. Also, the skin friction coefficient, reduced Nusselt number, and Sherwood number are computed numerically for various values of the physical parameters. It is found that the velocity becomes greater with an elevation in the value of the Helmholtz–Smoluchowski velocity. Meanwhile, it enlarges with rising in the value of the electro-osmotic parameter. The rise in the value of the thermal radiation parameter causes a dwindling influence on both temperature and nanoparticles concentration. Investigations of these effects together are very useful due to their important vital applications in various scientific fields, especially in medicine and medical industries, such as endoscopes, respirators, and diverse medical implementations, as nanoparticles can be utilized in the remedy of cancer tumors. Additionally, electroosmotic flow is important due to its ability to control fluid movement and enhance mass transport, making it valuable in various application such as sample separation, drug delivery, and DNA analysis, offering enhanced efficiency and sensitivity.

## Introduction

Nanofluid is a traditional liquid consistingof small particles of a diameter lower than 100 nm. It can be defined as a kind of fluid having the distinctive ability to improve the fluid thermal properties. Nanofluids have many applications in medicine, industry, and engineering.Choi^1^ tested that the thermal conductivity of the base fluid will be improved after adding a small amount of these nanoparticles. Tripathi et al.^2^ studied on peristaltic flow of nanofluids, and they ensure that the nanofluids yield suppressed back flow compared with Newtonian fluids. MHD transport of a third-grade nanofluid through a porous medium in the presence of thermal diffusion and diffusion thermo effects is discussed byEldabe et al.^3^. Mekheimer et al.^4^ analyzed the blood flow with gold nanoparticles in thecatheter. The flow behavior of a pseudo-plastic fluid containing tri-hybrid nanoparticles within the suspension; the flow is in the presence of different external effects such as Buoyancy forces, heat generation and viscous dissipations is discussed by Sohail et al.^5^. Nazir et al.^6^ examined the flow characteristics of a hyperbolic tangent liquid, considering the incorporation of ternary hybrid nanoparticles; the study analyzed the flow under various influencing factors, including a non-Darcy porous medium, surface rotation, external magnetic field, heat generation, and viscous dissipations. Many researchers have studied nanofluids flow through different surfaces^7–19^.

The study of non-Newtonian fluids is considered to be highly significant in engineering and applied science fields. There are various rheological models which utilizing to analyze and display the features of flow and transfer of heat. Although this model presents considerable mathematical complexity, it has garnered significant attention due to several compelling factors. Firstly, its constitutive relationship is established empirically, providing a practical approach. Secondly, the Eyring-Powell model exhibits both Newtonian behavior under both low and high shear stresses, making it particularly noteworthy. This model in the presence of different external forces plays an essential role in natural and geophysical processes which include delivery of dampness and temperature over environmental pollution, damaging of crops due to freezing, underground energy transport, geothermal reservoirs, thermal insulation, and agricultural fields^20–24^.

The phenomenon of both heat and mass transfer plays a significant role in various industrial and engineering processes, such as equipment power collectors, food processing, heat exchangers, damage of crops, refrigeration, and reservoir engineering in connection with thet hermal recovery process. So, in literature, convectional transport theories for heat and mass are utilized by several researchers.The flow phenomenon in this case is relatively complex because theseprocesses are containing heat transfer in non-Darcy porous media.Moreover, in the study of the dynamics of hot and salty springs of a sea. Fourier^25^ is the first who introduce the heat conduction law and heat transfer properties. The electromagnetic field and Biot number effects on non-Newtonian nanofluid flow with heat transfer through a non-Darcy porous medium are analyzed by Abouzeid^26^. Ismael et al.^27^ discussed the effect of temperature conditions, slip velocity, and entropy generation on MHD biviscosity micropolar nanofluid flow via a porous medium in a peristaltic channel. The flow of non-Newtonian fluid past a shrinking plate through a porous media with transferring heat and mass is explained by Eldabe et al.^28^. Several investigators discussed the flow with the impact of heat transfer of nanofluid^29–34^.

Electro-osmosis force (EOF) is due to the electrolyte solution flow under the effect of an external electric field on an ionized certain surface. The surface catches ions of the opposite sign from the electrolyte solution and holds the ions of the same sign to generate an electric double layer (EDL). In this case, electro-osmotic flow can be generated in combination with an electrolyte and an insulating solid. In addition, in natural unfiltered water, as well as buffered solutions, electro-osmotic flow can occur. The electro-osmosisexternal force isfirst studied by Reuss^35^. MHD peristaltic flow of Jeffery fluid through micro annulus in the presence of electro-osmosis force was studied by Mekheimer et al.^36^. Nadeem et al.^37^ observed electro-osmosis force on the microvascular blood flow. The electro-osmosis force and chemical reaction effects on the peristaltic flow of non-Newtonian nanofluid are focused on by Hegazy et al.^38^.

As stated by the above studies, the fundamental target of this study is to describe the impacts of time- periodic variations as well as electro-osmosis forces on the flow of Eyring-Powel nanofluid through a non-Darcy porous media. The fluid is flowing past an infinite vertical plate under the effects of viscous dissipation, Soret with Dufour impacts, chemical reaction, and heat source.We transform the system of non-linear partial differential equations which govern the problem into algebraic non-linear equations by using the explicit finite difference method. Then, the numerical formulas for the velocity, temperature, and nanoparticles concentration as well as the skin friction, reduced Nusselt number, and Sherwood number are obtained. The influences of diverse physical parameters on the various distributions are computed numerically and displayed through a set of graphs.The computed numerical results are given using tables for parameters of engineering importance. Furthermore, there is a strong correlation seen between the current solutions and the earlier stated outcomes in the relevant circumstances. Physically, nanofluids has several implementations in diverse scientific fields like the medical industry; medicine. For example, some nanoparticles are utilized in the therapy of cancer tumors. Additionally, the current study will serve as a vehicle for understanding more complex problems in industry, engineering such as separation processes, flow tracers, polishing of prosthetic heart valves, reducing friction in oil pipelines, cooling of metallic plates, and other fields.

## Mathematical formulation

Eyring-Powell model^13,20^ is chosen to describe the non-Newtonian fluid, which is in the usual notation given as:

1 $$ \tau_{ij} = \left[ {\mu + \frac{1}{b}\sinh^{ - 1} \left( \frac{1}{a} \right)} \right]\frac{{\partial V_{i} }}{{\partial x_{j} }},\,\,\, \, $$

where $$\tau_{xx} = 2\mu \frac{\partial u}{{\partial x}} + \frac{1}{b}\sinh^{ - 1} \left( {\frac{2}{a}\frac{\partial u}{{\partial x}}} \right)$$,

$$ \tau_{xy} = \tau_{yx} = \mu \left( {\frac{\partial u}{{\partial y}} + \frac{\partial v}{{\partial x}}} \right) + \frac{1}{b}\sinh^{ - 1} \left( {\frac{1}{a}\left( {\frac{\partial u}{{\partial y}} + \frac{\partial v}{{\partial x}}} \right)} \right), $$

$$ \tau_{yy} = 2\mu \frac{\partial v}{{\partial y}} + \frac{1}{b}\sinh^{ - 1} \left( {\frac{2}{a}\frac{\partial v}{{\partial y}}} \right). $$

**Figure 1:**
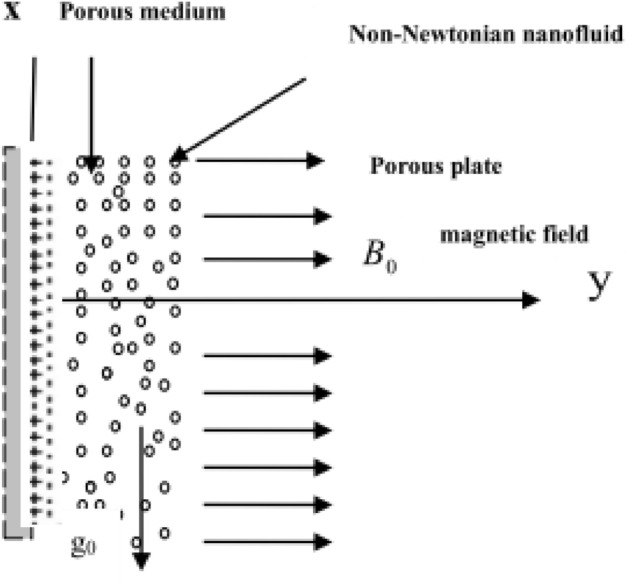
Sketch of the problem.

Consider the infinite vertical plate entrenched in an incompressible fluid (see Fig. 1). Initially, the temperature and nanoparticles concentration of both are assumed at $T_{\infty }$ and $C_{\infty }$. Then, at t > 0, the plate temperature and nanoparticles concentration are elevated to $T_{\omega }$ and $C_{\omega }$, and a periodic temperature and nanoparticles concentration are assumed. A uniform magnetic field B_0_ is applied transversally to the flow. We choose any point of the flat vertical infinite plate to be the origin of the coordinate system, the x − axis is chosen along the vertical plate vertically upwards, and the y − axis perpendicular to the plate. The following set of differential equations can be written by using boundary-layer assumptions as^6,8,13,20,29^:

2 $$ \frac{\partial u}{{\partial t}} = v\frac{{\partial^{2} u}}{{\partial y^{2} }} + \frac{1}{\rho \,a\,b}\left( {\frac{{\partial^{2} u}}{{\partial y^{2} }}} \right)\frac{1}{{\sqrt {\frac{1}{{a^{2} }}\left( {\frac{\partial u}{{\partial y}}} \right)^{2} + 1} }} - \left( {\frac{{\sigma B_{0}^{2} }}{\rho } + \frac{v}{K}} \right)\,u\, - \overline{b} \,u^{2} + \rho_{e} \,E_{x} + \,g_{0} \beta (T - T_{\infty } ) + g_{0} \beta^{*} (C - C_{\infty } ), $$

3 $$ \begin{aligned} \frac{\partial T}{{\partial t}} & = \frac{k}{{\rho \,C_{p} }}\frac{{\partial^{2} T}}{{\partial y^{2} }} + \frac{v}{{C_{p} }}\left( {\frac{\partial u}{{\partial y}}} \right)^{2} + \frac{1}{{\rho bC_{p} }}\left( {\frac{\partial u}{{\partial y}}} \right)\sinh^{ - 1} \left( {\frac{1}{a}\frac{\partial u}{{\partial y}}} \right)\, + \nabla \cdot \underline{q} \\ &\quad+ \frac{{\sigma B_{0}^{2} u^{2} }}{{\rho C_{p} }} + \,\frac{{D_{B} K_{T} }}{{C_{p} C_{s} }}\frac{{\partial^{2} C}}{{\partial y^{2} }} + Q_{0} \,(T - T_{\infty } ) + D_{B} \,\left( {\frac{\partial T}{{\partial y}}\,\frac{\partial C}{{\partial y}}} \right) + D_{T} \,\left( {\frac{\partial T}{{\partial y}}} \right)^{2} , \end{aligned} $$

4 $$ \frac{\partial C}{{\partial t}} = D_{B} \frac{{\partial^{2} C}}{{\partial y^{2} }} + \frac{{D_{T} }}{{T_{m} }}\frac{{\partial^{2} T}}{{\partial y^{2} }} - A(C - C_{\infty } )^{m} . $$

The appropriate initial and boundary conditions of the above equations may be expressed as^20,29^:

5a $$ u = 0,\,\,\,\,\,\,T = T_{\infty } ,\,\,\,\,\,\,C = C_{\infty } \,\,\,\,\,\,\,\,\,\,\,\,\,\,\,\,\,\,\,\,\,\,\,\,\,\,\,\,\,\,\,\,\,\,\,\,\,\,\,\,\,\,\,\,\,\,\,\,\,\,\,for\,all\,\,\,\,t \le 0, $$

5b $$ \begin{gathered} u = 0,\,\,\,\,\,T = T_{\infty } + \varepsilon \,(T_{\omega } - T_{\infty } )\cos \omega \,t,\,\,\, \hfill \\ \,\,\,\,\,\,\,\,\,\,\,\,\,\,\,\,\,\,C = C_{\infty } + \varepsilon \,(C_{\omega } - C_{\infty } )\cos \omega \,t\,\,\,\,\,\,\,\,\,\,\,\,\,\,\,\,\,\,\,\,\,\,\,\,\,\,at\,\,y = 0,\,\,\,\,\,t > 0, \hfill \\ \end{gathered} $$

5c $$ u \to 0,\,\,\,\,T \to T_{\infty } ,\,\,\,\,C \to C_{\infty } \,\,\,\,\,\,\,\,\,\,\,\,\,\,\,\,\,\,\,\,\,\,\,\,\,\,\,\,\,\,\,\,\,\,\,\,\,\,\,\,\,\,\,\,\,\,\,{\text{as}}\,\,\,\,\,y \to \infty ,\,\,\,\,t > 0, $$

By usingtheRosseland approximation^39^, the radiative heat flux may be defined as:

6 $$ q_{y} = \frac{{ - 4\sigma^{ * } }}{{3k_{R} }}\frac{{\partial T^{4} }}{\partial y}. $$

The temperature differences within the flow are small such that *T*^4^ may be expressed as a linear function of temperature. This is achieved by expanding *T*^4^ in a Taylor series about *T*_m_ and omitting higher-order terms^39^, one gets:

7 $$ T^{4} \approx 4T_{m}^{3} \,T - 3T_{m}^{4} . $$

By applyingGaussian’s law^14,36–38^, one gets:

8 $$ \nabla \cdot \underline{E} = \rho_{e} \,\overline{\varepsilon } , $$

We assume that the electric field is a conservative field^14,36–38^, then

9 $$ \underline{E} = - \nabla \varphi \,\,{\text{and therefore}},\,\,\nabla^{2} \varphi = - \rho_{e} \,\overline{\varepsilon } , $$

By using theBoltzmann distribution^14,36–38^, the net charge density can be written as:

10 $$ \rho_{e} = - Z_{\nu } \,e\,(n^{ - } - n^{ + } ), $$

11 $$ n^{ \pm } = n_{0} \, e^{{ \mp \frac{{e \, z_{\nu } }}{{k_{B} T_{av} }}\,\varphi }} , $$

According to the Debye–Huckel linearization principle $\frac{{e \, z_{\nu } }}{{k_{B} T_{av} }} \ll 1$, then, Eq. (10) became as follows:

12 $$ \rho_{e} = \frac{{\overline{\varepsilon } }}{{\lambda_{e}^{2} }}\varphi , $$

where $\lambda_{e} = \frac{1}{{e \, z_{\nu } }}\sqrt {\frac{{\overline{\varepsilon } \,k_{B} \,T_{av} }}{{2n_{0} \,}}}$ Then, according to boundary-layer assumption, Eq. (10) may be written as:

13 $$ \frac{{d^{2} \varphi }}{{dy^{2} }} = \frac{1}{{\lambda_{e}^{2} }}\varphi . $$

Let us introduce the following dimensionless quantities^6,13,14,20,29^:

14 $$ t^{ * } = \frac{t}{{t_{R} }},\,\,\,\,\omega^{ * } = t_{R} \,\omega ,\,\,\,\,y^{*} = \frac{y}{{L_{R} }},\,\,\,u^{*} = \frac{u}{{u_{R} }},\,\,\,T^{*} = \frac{{T - T_{\infty } }}{{T_{\omega } - T_{\infty } }},\,\,C^{*} = \frac{{C - C_{\infty } }}{{C_{\omega } - C_{\infty } }},\,\,\,\,\varphi^{*} = \frac{\varphi }{\xi }, $$

15 $$ {\text{where}}\,\,u_{R} = (\nu g_{0} \beta \Delta T)^{\frac{1}{3}} ,\,\,L_{R} = \left( {\frac{{g_{0} \beta \Delta T}}{{\nu^{2} }}} \right)^{{ - \frac{1}{3}}} \,\,{\text{and}}\,\,\,t_{R} = (g_{0} \beta \Delta T)^{{ - \frac{2}{3}}} \nu^{\frac{1}{3}} . $$

Then, Eq. (13) may be expressed as:

16 $$ \frac{{d^{2} \varphi }}{{dy^{2} }} = m_{e}^{2} \,\varphi . $$

By applying the boundary conditions $\varphi = 1\,\,\,\,at\,\,\,y = 0$ and $\varphi \to \infty \,\,\,\,at\,\,\,y \to \infty$, the analytical solution of Eq. (16) may be expressed as:

17 $$ \varphi = e^{{ - m_{e} \,y}} . $$

Then, the system of Eqs. (2), (3) and (4) is obtained in the dimensionless form as follows, after dropping the star mark

18 $$ \frac{\partial u}{{\partial t}} = \left( {1 + \frac{{\hat{\alpha }}}{{\sqrt {\gamma \,\left( {\frac{\partial u}{{\partial y}}} \right)^{2} + 1} }}} \right)\,\frac{{\partial^{2} u}}{{\partial y^{2} }}\, - \left( {M + \frac{1}{Da}} \right)\,u - Fs\,u^{2} + U_{HS} \,m_{e}^{2} \,e^{{ - m_{e} \,y}} + T + N\,C, $$

19 $$ \begin{aligned} \frac{\partial T}{{\partial t}} & = \frac{(3 + 4R)}{{3\Pr }}\frac{{\partial^{2} T}}{{\partial y^{2} }} + Ec\,\left( {\frac{\partial u}{{\partial y}}} \right)^{2} \, + \frac{Ec}{\delta }\,\left( {\frac{\partial u}{{\partial y}}} \right)\,\sinh^{ - 1} \left( {\sqrt \gamma \frac{\partial u}{{\partial y}}} \right)\, \\ &\quad+ M\,Ec\,u^{2} \, + \,Q_{0} \,T\, + Nb\,\left( {\frac{\partial T}{{\partial y}}\,\frac{\partial C}{{\partial y}}} \right) + Nt\,\left( {\frac{\partial T}{{\partial y}}} \right)^{2} + D_{f} \frac{{\partial^{2} C}}{{\partial y^{2} }}, \end{aligned} $$

20 $$ \frac{\partial C}{{\partial t}} = \frac{1}{Sc}\frac{{\partial^{2} C}}{{\partial y^{2} }} + \frac{Nt}{{Nb}}\,\frac{{\partial^{2} T}}{{\partial y^{2} }} - \delta \,C^{m} , $$

The initial and boundary conditions in the dimensionless form are

21a $$ u = 0,\,\,T = 0, \,\,C = 0{\text{ for}}\,\,{\text{all y}},{\text{ t}} \le 0, $$

21b $$ u = 0,\,\,T = \varepsilon \,{\text{cos }}\omega {\text{t}}, \,C = \varepsilon \,{\text{cos}}\,\,\omega {\text{t}} \,\,{\text{at y}} = 0, {\text{t}} > 0, $$

21c $$ {\text{and }}u \to 0, T \to 0, C \to 0 {\text{as}} {\text{y}} \to \infty , {\text{t}} > 0 $$

## The finite difference method

The governing Eqs. (18) → (20) and the boundary conditions (21a, 21b and 21c) are solved numerically by using a standard explicit finite–difference technique^16^. Here, we can write

22 $$ \begin{gathered} \frac{\partial u}{{\partial t}} = \frac{{u_{i}^{n + 1} - u_{i}^{n} }}{\Delta \tau } + O(\Delta \tau ),\,\,\,\,\,\,\,\,\,\,\frac{\partial T}{{\partial t}} = \frac{{T_{i}^{n + 1} - T_{i}^{n} }}{\Delta \tau } + O(\Delta \tau ), \hfill \\ \frac{\partial C}{{\partial t}} = \frac{{C_{i}^{n + 1} - C_{i}^{n} }}{\Delta \tau } + O(\Delta \tau ),\,\,\,\,\,\,\frac{\partial u}{{\partial y}} = \frac{{u_{i + 1}^{n} - u_{i}^{n} }}{\Delta y} + O(\Delta y), \hfill \\ \frac{{\partial^{2} u}}{{\partial y^{2} }} = \frac{{u_{i + 1}^{n} - 2\,u_{i}^{n} + u_{i - 1}^{n} }}{{(\Delta y)^{2} }} + O(\Delta y)^{2} , \hfill \\ \frac{{\partial^{2} T}}{{\partial y^{2} }} = \frac{{T_{i + 1}^{n} - 2\,T_{i}^{n} + T_{i - 1}^{n} }}{{(\Delta y)^{2} }} + O(\Delta y)^{2} , \hfill \\ \frac{{\partial^{2} C}}{{\partial y^{2} }} = \frac{{C_{i + 1}^{n} - 2\,C_{i}^{n} + C_{i - 1}^{n} }}{{(\Delta y)^{2} }} + O(\Delta y)^{2} , \hfill \\ \end{gathered} $$

where the indexi refers to *y* and the∆* y* = h = 0.05 and ∆$\tau$ = 0.003. According to the boundary conditions (21a, 21b and 21c), theMathematica package is used to solve Eqs. (18), (19) and (20) numerically, then a Newton iteration method continues until either the goals specified by accuracy goal or precision goal are achieved and determine the velocity and uniform magnetic field as afunction of y.

### Consistency of the finite difference scheme

The term consistency, which is applied to a finite difference method, means that the procedure may in fact approximate the solution of the partial differential equation of the present problem and not the solution of any other partial differential equation. The consistency is measured in terms of the difference between a differential equation and a difference equation. For consistency of Eqs. (18), (19) and (20), we estimate

23 $$ \begin{gathered} \left\{ {\frac{{u_{i}^{n + 1} - u_{i}^{n} }}{\Delta \tau } - \left( {\frac{{u_{i + 1}^{n} - 2u_{i}^{n} + u_{i - 1}^{n} }}{{(\Delta y)^{2} }}} \right)\left( {1 + \frac{{\hat{\alpha }}}{{\sqrt {\gamma \left( {\frac{{u_{i + 1}^{n} - u_{i}^{n} }}{\Delta y}} \right)^{2} + 1} }}} \right)\, + \left( {M + \frac{1}{{D_{a} }}} \right)\,u_{i}^{n} - Fs\,\left( {u_{i}^{n} } \right)^{2} + } \right. \hfill \\ \left. {U_{HS} \,m_{e}^{2} \,e^{{ - m_{e} \,i\Delta y}} - T_{i}^{n} - N\,C_{i}^{n} } \right\} - \left\{ {\frac{\partial u}{{\partial t}} - \left( {1 + \frac{{\hat{\alpha }}}{{\sqrt {\gamma \,\left( {\frac{\partial u}{{\partial y}}} \right)^{2} + 1} }}} \right)\,\frac{{\partial^{2} u}}{{\partial y^{2} }}\, - \left( {M + \frac{1}{Da}} \right)\,u - } \right. \hfill \\ \left. {Fs\,u^{2} + U_{HS} \,m_{e}^{2} \,e^{{ - m_{e} \,y}} + T + N\,C} \right\}_{i,n} = O(\Delta \tau ) + O(\Delta \eta ), \hfill \\ \end{gathered} $$

24 $$ \begin{gathered} \left\{ {\frac{{T_{i}^{n + 1} - T_{i}^{n} }}{\Delta \tau } - \frac{(3 + 4R)}{{3\Pr }}\left( {\frac{{T_{i + 1}^{n} - 2T_{i}^{n} + T_{i - 1}^{n} }}{{(\Delta y)^{2} }}} \right) - Ec\,\left( {\frac{{u_{i + 1}^{n} - u_{i}^{n} }}{\Delta y}} \right)^{2} \,\, - \,\,\frac{{E_{c} }}{\delta }\left( {\frac{{u_{i + 1}^{n} - u_{i}^{n} }}{\Delta y}} \right) \times } \right. \hfill \\ \,\sinh^{ - 1} \left( {\sqrt \gamma \left( {\frac{{u_{i + 1}^{n} - u_{i}^{n} }}{\Delta y}} \right)} \right)\, - MEc\,(u_{i}^{n} )^{2} \, - Q_{0} \,T_{i}^{n} - Nb\,\left( {\frac{{T_{i + 1}^{n} - T_{i}^{n} }}{\Delta y} \times \frac{{C_{i + 1}^{n} - C_{i}^{n} }}{\Delta y}} \right) - \hfill \\ \left. { - Nt\,\left( {\frac{{T_{i + 1}^{n} - T_{i}^{n} }}{\Delta y}} \right)^{2} - D_{f} \left( {\frac{{C_{i + 1}^{n} - 2C_{i}^{n} + C_{i - 1}^{n} }}{{(\Delta y)^{2} }}} \right)} \right\}\, - \left\{ {\frac{\partial T}{{\partial t}} - \frac{(3 + 4R)}{{3\Pr }}\frac{{\partial^{2} T}}{{\partial y^{2} }} - Ec\,\left( {\frac{\partial u}{{\partial y}}} \right)^{2} \, - } \right. \hfill \\ \left. {\frac{Ec}{\delta }\,\left( {\frac{\partial u}{{\partial y}}} \right)\,\sinh^{ - 1} \left( {\sqrt \gamma \frac{\partial u}{{\partial y}}} \right)\, - M\,Ec\,u^{2} \, - \,Q_{0} \,T\, - Nb\,\,\left( {\frac{\partial T}{{\partial y}}\,\frac{\partial C}{{\partial y}}} \right) - Nt\,\left( {\frac{\partial T}{{\partial y}}} \right)^{2} - D_{f} \frac{{\partial^{2} C}}{{\partial y^{2} }}} \right\} \hfill \\ = O(\Delta \tau ) + O(\Delta y), \hfill \\ \end{gathered} $$

25 $$ \begin{aligned} & \left\{ {\frac{{C_{i}^{n + 1} - C_{i}^{n} }}{\Delta \tau } - \frac{1}{Sc}\left( {\frac{{C_{i + 1}^{n} - 2C_{i}^{n} + C_{i - 1}^{n} }}{{(\Delta y)^{2} }}} \right) - \frac{Nt}{{Nb}}\left( {\frac{{T_{i + 1}^{n} - 2T_{i}^{n} + T_{i - 1}^{n} }}{{(\Delta y)^{2} }}} \right) + \delta \,\left( {C_{i}^{n} } \right)^{m} } \right\} \\ &\quad- \left\{ {\frac{\partial C}{{\partial t}} - \frac{1}{Sc}\frac{{\partial^{2} C}}{{\partial y^{2} }} - \frac{Nt}{{Nb}}\,\frac{{\partial^{2} T}}{{\partial y^{2} }} + \delta \,C^{m} } \right\} = O(\Delta \tau ) + O(\Delta \eta ). \end{aligned} $$

Here, R.H.S. of Eqs. (23), (24) and (25) represent truncation error as $\Delta \tau \to 0$ with $\Delta y \to 0$, the truncation error tends to zero. Hence our explicit scheme is consistent.

### Global error estimation

We useZadunaisky technique^4^, to calculate the global error estimation G. E. E., which can be explained in the following steps:

1. Interpolate the functions $u_{i}^{n}$,$T_{i}^{n}$ and $C_{i}^{n}$ withtheir first derivatives, where (i = 1,2,….., 6) from their values, name them P_i_ (i = 1,2,...,6), and interpolate the functions of $u^{\prime\prime},\,T^{\prime\prime}\,\,{\text{and}}\,C^{\prime\prime}$, and name them as ${\text{R}}_{{1}} (y) = u^{\prime\prime},\,\,\,{\text{R}}_{{2}} (y) = T^{\prime\prime},\,\,\,{\text{R}}_{{3}} (y) = \,\,C^{\prime\prime}$.
2. Calculate the detect functions D_i_ (i = 1,2,…,6), which can be written as follows:
3. D_1_(*y*) = $${\text{P}}_{1}^{\prime } - {\text{P}}_{2}$$ = 0, D_2_(*y*) = $${\text{P}}_{2}^{\prime } - {\text{R}}_{{1}} (y)$$,
4. D_3_(*y*) = $$P_{3}^{\prime } - {\text{P}}_{4}$$ = 0, D_4_(*y*) = $${\text{P}}_{4}^{\prime } - {\text{R}}_{{2}} (y)$$,
5. D_5_(*y*) = $$P_{5}^{\prime } - {\text{P}}_{6}$$ = 0, D_6_(*y*) = $${\text{P}}_{6}^{\prime } - {\text{R}}_{{3}} (y)$$,
6. Add the detect functions Di (i = 1,2,…,6) to the original problems and replace every Yi by another variable Zi (i = 1,2,...,6).
7. Solve the pseudo -problem by the same method to get the solution $Z(z)$ whose elements $Zi$ (i = 1,2,…,6).
8. Calculate the global error from the relation en = Zn-P(zn), (n = 1,2,…,6), where Zn is the approximate solution of the pseudo -problemat the point $zn$ and $Z(zn)$ is the exact solution of the pseudo -problemat $zn$. Obviously, the exact solution of pseudo –problemis. Z(z_n_)= P(z_n_).
9. The values of the global error are presented in Table 1. This error is based on using 11 points to find the interpolating polynomials PI (I = 1, 2,… 6), of degree 10^4^.

**Table 1:** The values of the global error estimation for the velocity, temperature and nanoparticles concentration.

Global error estimation						
*y*	*u*	e_1_	*T*	e_3_	*C*	e_5_
0	0	0	0.05	0	0.05	0
0.4	0.05786	0.290D–3	0.1215	0.500D–5	− 0.0115	− 0.100D–5
0.8	0.06072	0.400D–3	0.1351	0.700D–5	− 0.0186	− 0.500D–6
1.2	0.47400	0.400D–3	0.1084	0.700D–5	− 0.0065	− 0.500D–6
1.6	0.02607	0.290D–3	0.0605	0.500D–5	− 0.0003	− 0.100D–5
2	0	0	0	0	0	0

In order to achieve the above task we use the Mathematica package 10.1.

## The skin-friction,heat and mass transfer^40^

The skin-frictioncoefficient reduced Nusselt number and Sherwood number in the non-dimensional form can be written as:

26 $$ \tau_{\omega } = \left[ {\frac{{\partial^{2} u}}{{\partial y^{2} }}\left( {1 + \frac{\alpha }{{\sqrt {\gamma \left( {\frac{\partial u}{{\partial y}}} \right)^{2} + 1} }}} \right)} \right]_{y = 0} , $$

27 $$ Nu = - \left[ {\frac{\partial T}{{\partial y}}} \right]_{y = 0} , $$

28 $$ Sh = - \left[ {\frac{\partial C}{{\partial y}}} \right]_{y = 0} . $$

We can write equations (26), (27) and (28) by using finite difference method as follows:

29 $$ \tau_{\omega } = \left( {\frac{{u_{2}^{n} - 2u_{1}^{n} + u_{0}^{n} }}{{(\Delta y)^{2} }}} \right)\,\left( {1 + \frac{\alpha }{{\sqrt {\gamma \left( {\frac{{u_{1}^{n} - u_{0}^{n} }}{\Delta y}} \right)^{2} + 1} }}} \right), $$

30 $$ Nu = - \left( {\frac{{T_{1}^{n} - T_{0}^{n} }}{\Delta y}} \right), $$

31 $$ Sh = - \left( {\frac{{C_{1}^{n} - C_{0}^{n} }}{\Delta y}} \right). $$

## Results and discussion

In this section, we show the effects of the problem'sphysical parameters on the velocity of the fluid, temperature, nanoparticles concentration, skin frictioncoefficient, reduced Nusselt number, and Sherwood number. These impacts were evaluated by setting the following standard values:

$$\omega =0.005, \gamma =0.4, \varepsilon =0.05, , , M=10, Da=0.1,Fs=0.5, Gr=0.5,Pr=2.5, Q=5, Ec=1, Sc=1, Df=0.05, Nb=2.5, Nt=3.5,,$$$$U_{HS} = 2$$, R = 1, m = 1, and $$\delta = 0.1$$.

**Figure 2:**
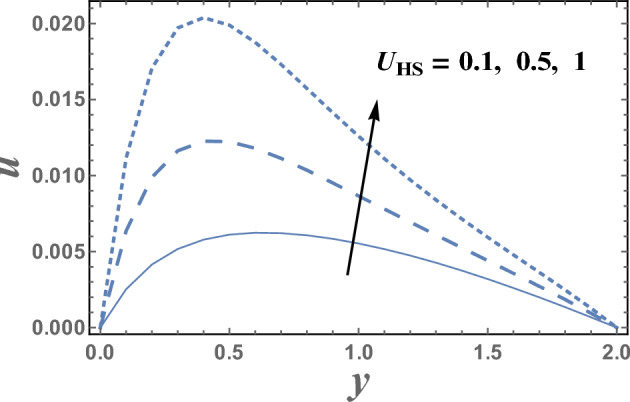
The velocity u is plotted with y, for different values of U_HS_.

**Figure 3:**
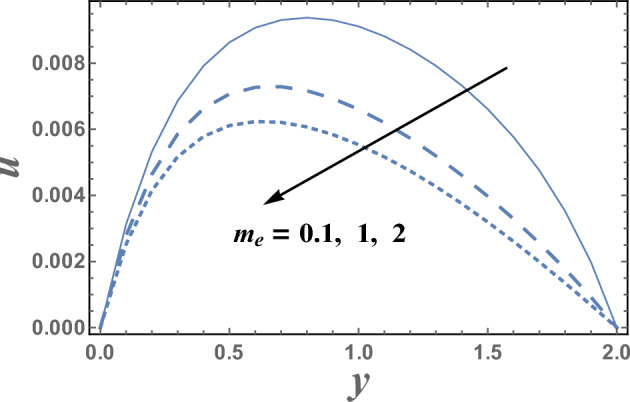
The velocity u is plotted with y, for different values of m_e_.

**Figure 4:**
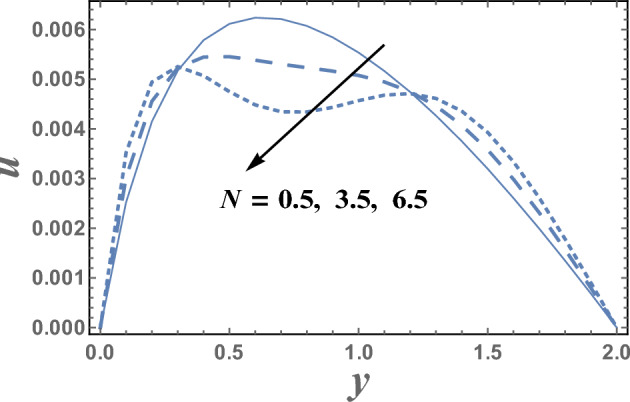
The velocity u is plotted with y, for different values of N.

**Figure 5:**
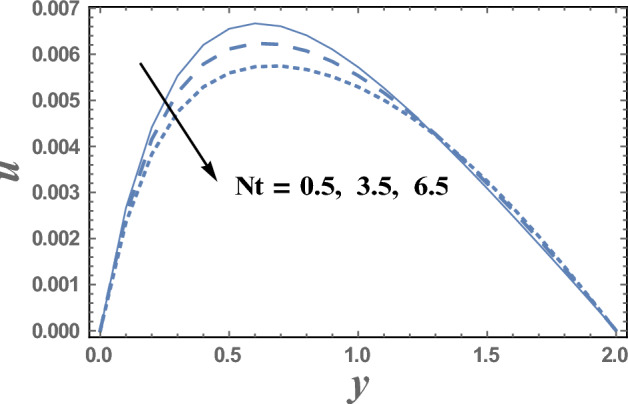
The velocity u is plotted with y, for different values of Nt.

Figures 2 and 3 are plotted to illustrate the influence of both theHelmholtz–Smoluchowski velocity dimensionless $$U_{HS}$$ and the electro-osmotic parameter $$m_{e}$$ on the velocity distribution $$u(y)$$. It is observed that the velocity distribution increases with an increase in the value of $$U_{HS} .$$ Meanwhile, it decreases as $$m_{e}$$ increases.Physically, Coulomb force induced by an electric field charges in a solution causes electro-osmotic flow. Because the chemical balance between a surface and an electrolyte solution usually leads to the interface acquiring a net fixed electrical charge, a layer of mobile ions, known as the Debye layer, creates in the region near the interface. When an electric field is applied to the fluid, the net charge in the electrical double layer is induced to move by the resulting Coulomb force. The resulting flow is termed electro-osmotic flow. So, the bigger resulting Coulomb force and consequently easily fluid flow. It is also noted that for each value of both $$U_{HS}$$ and $$m_{e}$$, there exists a maximum value of uand all maximum values occur at $$y \simeq 0.49$$. Figure 4 illustrates the effect of the buoyancy ratio*N*on the velocitydistribution u. It is found that the velocity increases withan enlargement in*N*in the intervals $$y\in \left[0.0, 0.3\right]\cup \left[1.2,\right).$$ otherwise it decreases by increasing *N*. So, the behavior of u in the interval $$y \in [0.3, 1.2]$$, is an inversed manner of its behavior in the other intervals. In this case, for each value of *N*, there are maximum values of u hold at $$y = 0.65.$$ Figure 5 illustrates the impact of the thermophoresis parameter $$Nt$$ on the velocity distribution $$u(y)$$. It is depicts that in the interval of the coordinate $$y[0.0, 1.29]$$, the behavior of u for various values of $$Nt$$ is exactly similar to the behavior of u for various values of $$m_{e}$$ given in Fig. 3. It is also noted, from Fig. 5, that in the interval of the radial coordinate $$y[1.29, )$$, the behavior of u is an inversed manner of its behavior in the $$y[0.0, 1.29]$$ except that the curves are very close to each other in the second interval. Moreover, in the first interval, there is a maximum value of u holds at $$y =0.62$$ and this maximum value slightly increases with an elevation in the value of $$Nt$$.

**Figure 6:**
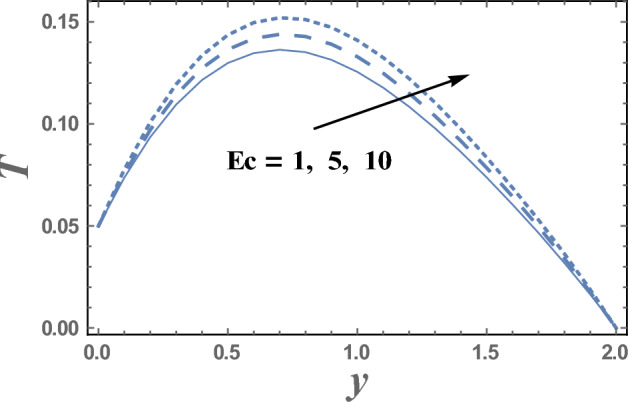
The temperature T is plotted with y, for different values of E_c_.

**Figure 7:**
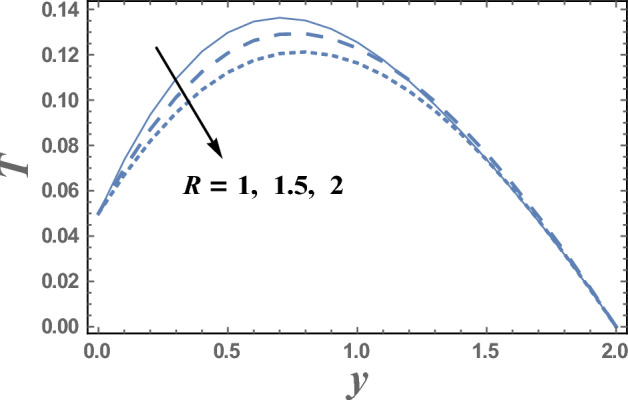
The temperature T is plotted with y, for different values of R.

**Figure 8:**
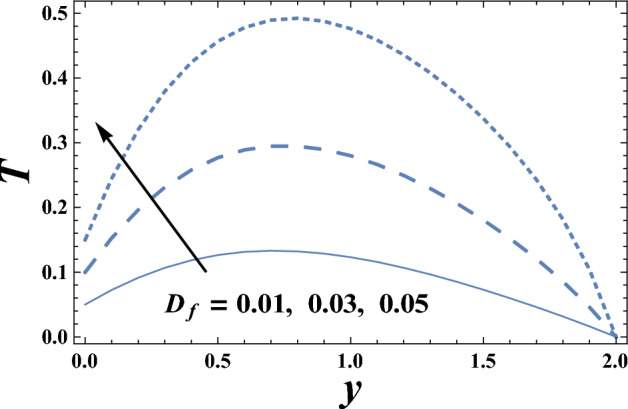
The temperature T is plotted with y, for different values of D_f_.

The variations of the temperature distribution T with the dimensionless coordinate y for various values ofboth Eckert number $Ec$ and radiation parameter R are displayed throughout Figs. 6 and 7 respectively. The graphical results of Figs. 6 and 7 indicate that the temperature distribution T increases with an increase in the parameter Ec.From the physical point of view; during the motion of the fluid particles, the fluid viscosity converts some kinetic energy into thermal energy. This process is called viscous dissipation because it occurs due to viscosity. So, viscous dissipation can be defined as a heat source that results from the irreversible work done by the fluid flow to conquer the shear forces layers in the flow and appears as an increase in the fluid temperature. Consequently, it interprets the result in Fig. 6. This behavior is in agreement with that reported by^40,41^. Meanwhile, it declineswith an enhancement in the value ofR. It is also noted that T increases with y till a definite value y = y_0_ (represents the maximum value of T) and it decreases afterward. Similarly, we draw the variation of *T* with *y* for different values of the thermophoresis parameter D_f_ in Fig. 8, we will obtain a figure in which the behavior of the curves is the same as that obtained in Fig. 6, with the only difference that the obtained curves are very larger to each other than those obtained in Fig. 6. The results in Fig. 8, is due to the following; the Dufour effect is the energy flux due to a mass concentration gradient occurring as a coupled effect of irreversible processes. It is the reciprocal phenomenon to the Soret effect^2^. The concentration gradient results in a temperature change. So, it always makes to increase the energy of luiquids.

**Figure 9:**
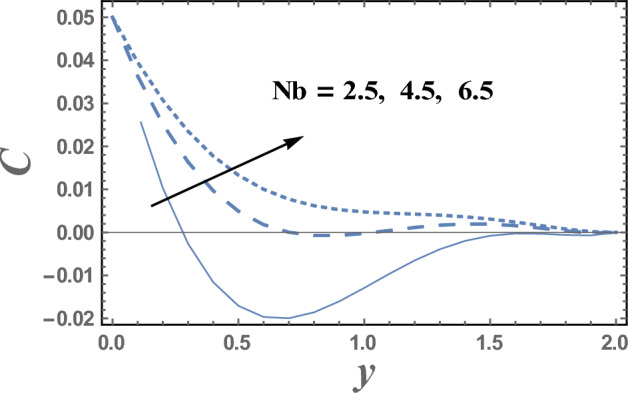
The nanoparticles concentration C is plotted with y, for different values of Nb.

**Figure 10:**
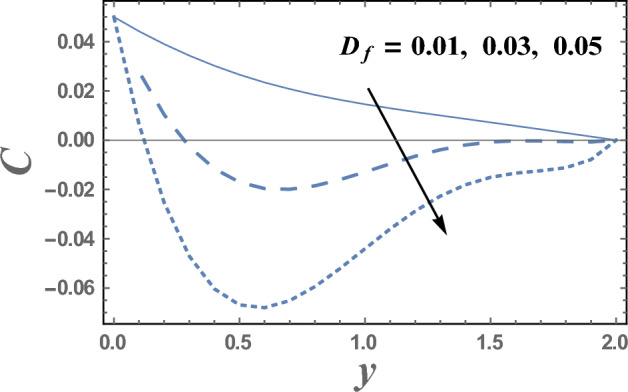
The nanoparticles concentration C is plotted with y, for different values of D_f_.

**Figure 11:**
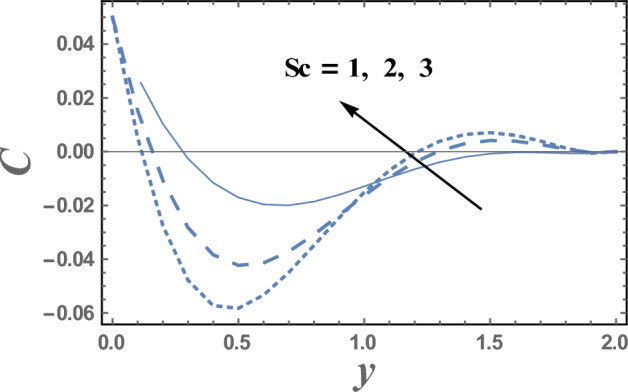
The nanoparticles concentration C is plotted with y, for different values of S_c_.

**Figure 12:**
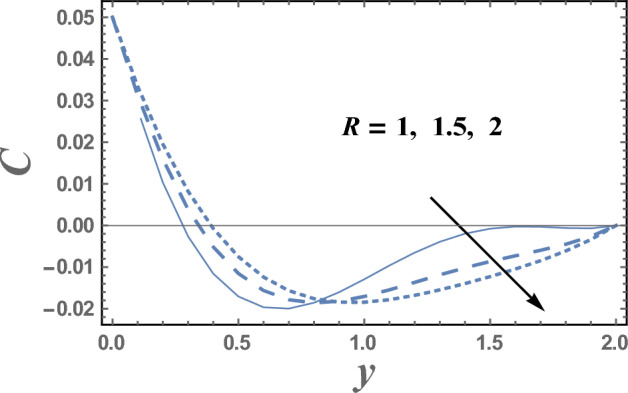
The nanoparticles concentration C is plotted with y, for different values of S_c_.

Brownian motion as a natural phenomenon, is the random motion of particles suspended in a mediumwhich may be a liquid or gas. This motion pattern typically consists of random fluctuations in a particle's position inside a fluid sub-domain, followed by a relocation to another sub-domain. Each relocation is followed by more fluctuations within the new closed volume. This pattern describes a fluid at thermal equilibrium and makes to increase the nanoparticles concentration. This will clarify the next result. Figures 9 and 10 represent the behaviors of the nanoparticles concentration distribution C with the dimensionless coordinate y for different values of Brownian motion parameter Nb and Dufour number D_f_, respectively. It is observed from Figs. 9 and 10, that the nanoparticles concentration enhances with the increase of Nb. Whereas it dwindles as $Df$ elevates, respectively. On the other hand, it had an inverse effect near the wall y = 0, namely in the interval y ∈ [0.0, 0.18]. It is also noted that the difference of the nanoparticles concentration distribution C for different values of Nb and Df, becomes lower with increasing the coordinate y and reaches minimum value, after which it increases. Figure 11 illustrates the effect of Schmidt number Sc on the nanoparticles concentration distribution C as a function of the dimensionless coordinate y. It is found that in the interval of the radial coordinate y $\in$[0.0, 1.1], the behavior of C for various values of Sc is exactly similar to the behavior of C for various values of Nb given in Fig. 9. It is also noted, from Fig. 11 that in the interval of the coordinate y $\in$[1.1, $\infty$), the behavior of C is an inversed manner of its behavior in the interval y $\in$[0.0, 1.1], except that the curves are very close to each other in the first interval. In this case, for any value of the parameter Sc, there is a minimum value of $C$ holds at y = 0.49, and this minimal slightly decreases by increasing the value of Sc. The influence of the thermal radiation parameter R on the nanoparticles concentration distribution C is illustrated in Fig. 12. It is found that the effect of R on C is opposite to the impact of $Sc$ on C given in Fig. 11, with the only difference that, the curves in Fig. 11 are very close to those to each other in the first interval than those obtained in Fig. 12. Now, we will explicate how the radiation parameter affects the nanoparticles concentration. The thermal radiation parameter is defined as the relative contribution of conduction heat transfer to thermal radiation transfer. It is evident that an increase in the radiation parameter causes in decreasing the nanoparticles concentration within the layer. The impacts of other parameters are similar tothat obtained in Figs. 9 and 10. But, they are excluded here to avoid any kind of repetition.

**Figure 13:**
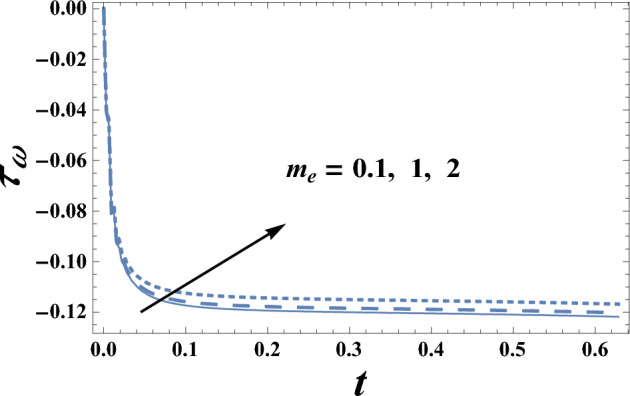
The skin friction coefficient $\tau_{\omega }$ is plotted with t, for different values of m_e_.

**Figure 14:**
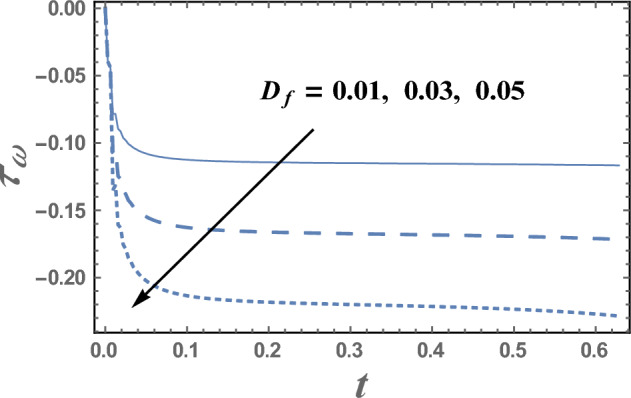
The skin friction coefficient $\tau_{\omega }$ is plotted with t, for different values of D_f_.

Figures 13 and 14 illustrate the behavior of skin friction coefficient $\tau_{\omega }$ with the time t, for various values of the electro-osmotic parameter $m_{e}$ and Dufour number D_f_. It is observed from these figures that skin friction coefficient increases as $m_{e}$ increases, while it decreases with the increase of D_f_. Moreover, we can notice from Figs. 13 and 14 that skin friction coefficient is always negative and decreases as t increases and at a finite value of t, the relation between $\tau_{\omega }$ and t is a straight line parallel to the time.

**Figure 15:**
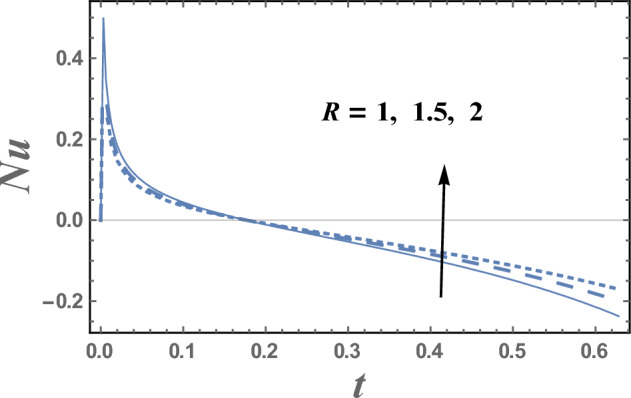
Nusselt number Nu is plotted with t, for different values of R.

**Figure 16:**
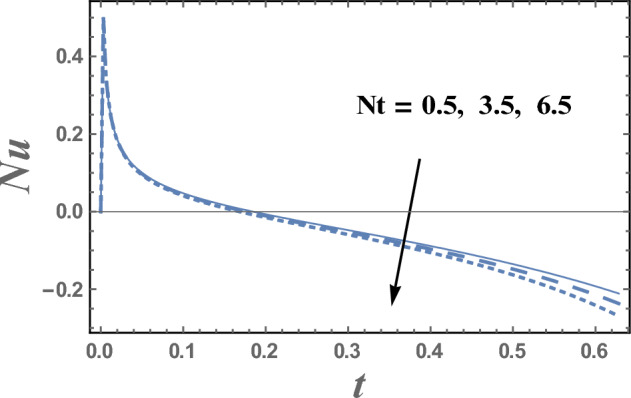
Nusselt number Nu is plotted with t, for different values of Nt.

The values of Nusselt number Nu are plotted versus the time t through Figs. 15 and 16 for various values of the thermal radiation parameter R and the thermophoresis parameter Nt. It is indicated from Figs. 15 and 16 that Nusselt number increases with increasing R and decreases with increasing values of R for $0 < \,t\, < 0.13$, while Nusselt number decreases as Nt increases. In addition, the values of Nu for different values of R and Nt, initially increases as t increases till a finite value of t, after which it crumbles.

**Figure 17:**
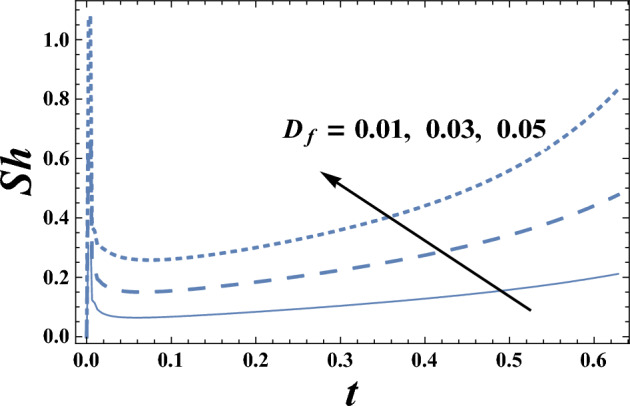
Sherwood number Sh is plotted with t, for different values of D_f_.

**Figure 18:**
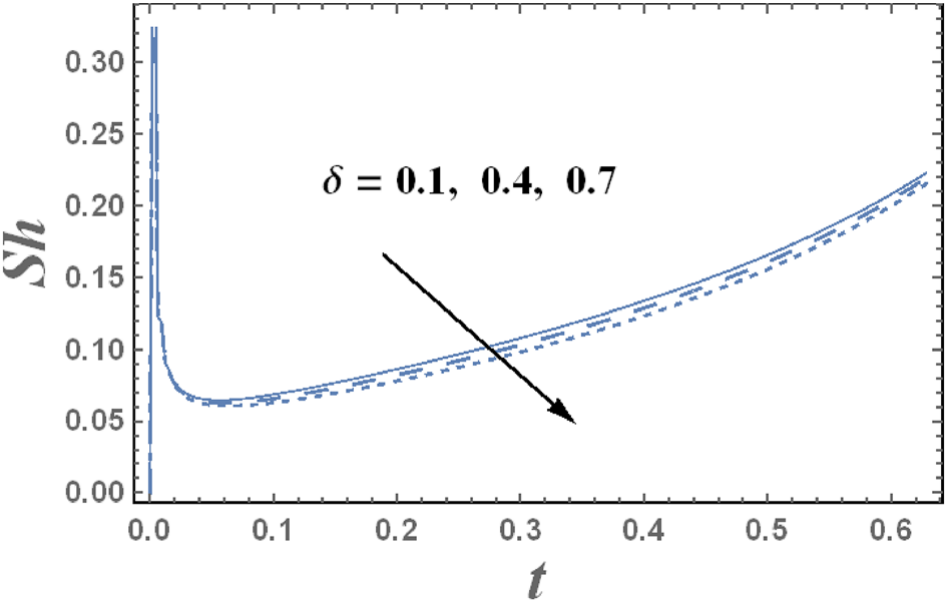
Sherwood number Sh is plotted with t, for different values of $\delta$.

The behavior of Sherwood number $Sh$ with the time t for various values of Dufour number D_f_, and the chemical reaction parameter $\delta$ are presented in Figs. 17 and 18, respectively. It is clear from these figures that Sherwood number decreases by increasing the chemical reaction parameter $\delta$, while it increases by increasing Dufour number D_f_. Moreover, it is noted that the difference of Sherwood number $Sh$ for different values of $\gamma$, and $Bn$ becomes lower with increasing t and reaches the minimum value, after which it increases. Note that the minimum value of Sherwood number decreases by increasing $\delta$, whereas it increases with the increase of D_f_. Further, it is found for each value of $\gamma$, $M$ $Bn$ and, $Sh$ is always positive.

**Figure 19:**
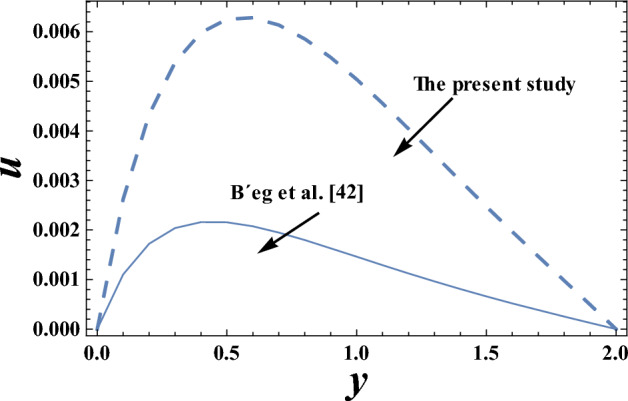
Comparison of the velocity values in our study and those obtained by B´eg et al.^42^.

**Figure 20:**
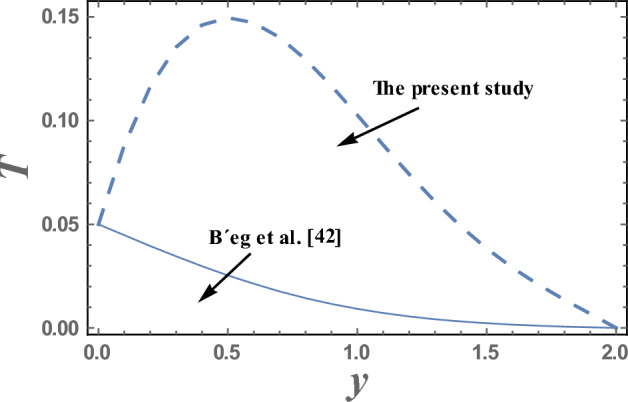
Comparison of the velocity values in our study and those obtained by B´eg et al.^42^.

Figures 19 and 20 display a comparison for the velocity and temperature values between our results and those obtained by B´eg et al.^42^. It is noticed that there is a good agreement in the obtained results.

Table 2 presents numerical results for the skin friction $\tau_{\omega }$, reduced Nusselt number *Nu* and Sherwood number *Sh*, for various values of the wave amplitude $\varepsilon$, Prandtl number $Pr$ and heat source parameter Q_0_^40^. It is clear from Table 2, that an increase in $\varepsilon$, $Pr$ and Q_0_ gives an increase in the values of dimensionless quantity $\tau_{\omega }$ and $Sh$, but decreasing in the dimensionless quantity *Nu*. In addition, these results have been compared with those obtained by B´eg et al.^42^, and it is found that there is a good agreement between our results and B´eg et al.^42^.

**Table 2:** Comparison between numerical results for the skin friction, reduced Nusselt number and Sherwood number in the present study and those obtained by B´eg et al.^42^.

$\varepsilon$	*P*_r_	*Q*_0_	$\tau_{\omega }$ in the present study	$\tau_{\omega }$(B´eg et al.^42^)	*Nu* in the present study	*Nu* (B´eg et al.^42^)	*Sh* in the present study
0.05	2.5	1	0.0797		− 0.0024		0.0066
0.1	2.5	1	0.1816		− 0.0083		0.0821
0.2	2.5	1	0.6342		− 0.1749		0.5998
0.2	3	1	0.2951	− 0.1867	− 0.0221	0.0957	0.2042
0.2	3.5	1	0.2954	− 0.0891	− 0.0320	0.0118	0.2166
0.2	4	1	0.2957	− 0.0107	− 0.0424	0.0083	0.2297
0.2	4	2	0.2290		− 0.2348		0.4089
0.2	4	3	0.3047		− 0.5515		0.6691
0.2	4	4	0.3169		− 1.1642		1.1732

## Conclusion

The main target of this study is to present the effects of electro-osmosis forces on the free convective flow of Eyring-Powel nanofluid through a non-Darcy porous medium. The system is influenced by an external uniform magnetic field, thermal radiation, heat source, Ohmic dissipation,and viscous dissipation. The explicit-finite difference method is applied to obtain a numerical solution to the equations that govern the fluid motion. In addition, we obtain an estimation of the error propagation by using Zadunaisky technique for the finite difference method. The estimated errors ensure the usage of the approximated solutions as a suitable approximation to the calculated physical values. It is hoped that the present work will serve as a vehicle for understanding more complex problems in industry, engineering, and some physiological flows^43–46^. The obtained results are also shown in a graphic representation and can be summarized as follows:

1. The velocity *u* decreases with anenrichin $\upvarepsilon$ $m_{e}$, M, R, $Sc$ and $\alpha$.Whilst it elevates as Da,$\varepsilon$, $Nb$ and *Q*_0_ increase. In addition, as both N and $Nt$ increase, it increases or dwindles.
2. By increasing the coordinate y, the velocity *u* for different values of problem physical parameters becomes greater and reaches a maximum value at a finite value, after which, it declines.
3. The temperature T increases with an enhancement inthe values of $Nt, Nb, Df, Q0$ and* Ec.*Whereas it dwindles or elevates as both R and $Pr$ enhance.
4. All curves of the temperature for different values of the several physical parameters don’t intersect at the plate $y=0$, then increase as y escalates till a maximum value.
5. The behavior of nanoparticles concentration C seems to be opposite to the temperature behavior.

### Future perspectives

Numerous potential applications in bioinformatics, fluid dynamics problems, and critically important financial mathematics may be numerically treated using the Lobatto IIIA and spectral collocation algorithms.

## Data Availability

The datasets generated and/or analyzed during the current study are not publicly available due [All the required data are only with the corresponding author] but are available from the corresponding author on reasonable request.

## Roman symbols

a and b: Parameters of Eyring-Powell model
$\overline{b}$: Non-Darcian parameter
A: Chemical reaction parameter
B_o_: Strength of uniform magnetic field
C: Nanoparticles concentration
C_E_: Ergun constant
C_p_: Specific heat at constant pressure
C_s_: Concentration susceptibility
Da: Darcy number
D_f_: Dufour number
*D*_B_: Brownian diffusion coefficient
*D*_T_: Thermophoretic diffusion coefficient
e: The electronic charge
Ec: Eckert number
E_x_: The electric field component
Fs: Forchheimer number
g_0_: Gravity acceleration
k: Thermal conductivity
K: Permeability constant
K_B_: Boltzmann constant
K_T_: Thermal diffusion ratio
m: Chemical reaction order
m_e_: The electroosmotic parameter
M: Magnetic parameter
n_0_: Bulk nanoparticles concentration
n^+^: The cations
n^–^: The anions
N: Buoyancy ratio
Nb: Brownian motion parameter
Nt: The thermophoresis parameter
Pr: Prandtl number
q: The radiative heat flux
Q_0_: Heat source parameter
R: The radiation parameter
Sc: Schmidt number
t: Time
T: Temperature of the fluid
T_av_: The average temperature
T_m_: The mean temperature
U_HS_: Helmholtz Smoluchowski velocity
V_i_: Velocity vector = (u(y, t), 0, 0)
$Z_{\nu }$: The charge balance

## Greek symbols

$\hat{\alpha }$: Non dimensional parameter
β: Volumetric coefficient of thermal expansion
β^*^: Volumetric of expansion with nanoparticles concentration
δ: Non dimensional parameter
$\overline{\varepsilon }$: The dielectric permittivity
$\varphi$: The electric potential
γ: Non dimensional parameter
μ: Viscosity
ν: Kinematic viscosity
ω: Frequency of the oscillating plate
ρ: The fluid density
ρ: The fluid density
$\rho_{e}$: The total ionic energy density
σ: Electrical conductivity of the fluid
$\tau$: Stress tensor in Eyring-Powell model
$\xi$: The mean of electric potential

## Superscripts and subscripts

∞: Free stream condition
w: Wall or plate condition
